# Comprehensive Genomic Characterization of m^6^A Methylation Machinery and Its Cadmium-Responsive Expression Profiles in Pepper (*Capsicum chinense*)

**DOI:** 10.3390/ijms27094110

**Published:** 2026-05-04

**Authors:** Hao Xu, Wei Li, Yiwen Wang, Wenlong Bao

**Affiliations:** 1 School of Breeding and Multiplication (Sanya Institute of Breeding and Multiplication), Hainan University, Sanya 572025, China; haoxu@hainanu.edu.cn (H.X.);; 2 Key Laboratory for Quality Regulation of Tropical Horticultural Crops of Hainan Province, School of Tropical Agriculture and Forestry (School of Agriculture and Rural Affairs, School of Rural Revitalization), Hainan University, Danzhou 571737, China

**Keywords:** epitranscriptomics, RNA methylation, cadmium stress, genotype-dependent response, gene expression

## Abstract

N6-methyladenosine (m^6^A) is a dynamic and reversible RNA modification governed by the tripartite machinery of writers, erasers, and readers, which plays crucial roles in plant adaptation to environmental stresses. However, the repertoire and function of m^6^A machinery in pepper (*Capsicum chinense*) remain uncharacterized. Here, we performed a genome-wide identification of m^6^A regulators in pepper (*Ccm^6^As*), revealing 20 high-confidence genes, comprising 6 writers, 7 erasers, and 7 readers. Comprehensive analysis of their phylogeny, conserved domains, and promoter *cis*-elements revealed structural conservation and a transcriptional architecture highly enriched in stress-responsive elements. Notably, in silico analysis revealed that the core catalytic writer CcMTA, the essential adaptor CcFIP37A, and the paralog CcMTB1 exhibited strong interactions, suggesting the formation of a functional methyltransferase complex. Using a comparative approach with cadmium-tolerant (CdRes-1) and cadmium-sensitive (CdSen-1) genotypes, we found that Cd stress induced a genotype-specific transcriptional reprogramming of the m^6^A machinery. In the tolerant genotype CdRes-1, eight regulators were significantly upregulated, whereas only *CcMTA* showed a modest induction in the sensitive CdSen-1. Subcellular localization experiments confirmed dual nuclear and cytoplasmic localization of CcMTA. Our findings provide a foundational epitranscriptomic resource for future functional investigations into the molecular mechanisms underlying cadmium stress responses in pepper.

## 1. Introduction

N6-methyladenosine (m^6^A) is the most prevalent internal chemical modification in eukaryotic messenger RNA (mRNA). This dynamic and reversible post-transcriptional regulatory mechanism operates through three classes of proteins (writers, erasers, and readers) that collectively mediate its biological functions. The writer complex, such as METTL3/METTL14/WTAP in mammals, recognizes the RRACH motif and catalyzes methylation, whereas erasers like FTO and ALKBH5 remove methyl groups, thereby maintaining m^6^A’s dynamic balance [1,2]. These m^6^A modifications are specifically recognized by reader proteins, including YTH-domain family members, which subsequently regulate mRNA splicing, nuclear export, stability, translation, and decay [2]. Through this sophisticated “writer–eraser–reader” network, m^6^A modification acts as a key post-transcriptional regulator, broadly participating in gene expression control, cellular homeostasis, and organismal responses to environmental stimuli [3,4].

In plants, the m^6^A modification system is evolutionarily conserved but exhibits species-specific variations in nomenclature and function [5]. Widely found in species such as *Arabidopsis*, rice, and tomato, m^6^A modification is dynamically regulated through the coordinated action of writers (multi-subunit complexes containing MTA, MTB, FIP37, VIR, and HAKAI), erasers (e.g., the demethylase ALKBH10B), and readers (YTH-domain proteins such as ECT2/3/4) [6]. This system is essential throughout the entire plant life cycle. During early development, mutations in writer components *MTA*, *MTB*, or *FIP37* can lead to embryonic lethality or severe developmental defects [7]. In the vegetative phase, reader proteins ECT2/3/4 regulate leaf morphogenesis [8]. The floral transition is modulated by ALKBH10B-mediated demethylation of flowering-related genes such as *FLOWERING LOCUS T* (*FT*) [9]. Moreover, m^6^A plays a critical role in plant stress responses. For instance, the m^6^A reader protein ECT8 in *Arabidopsis* acts as a key “stress sensor”: it senses salt-stress signals, upregulates its expression, and accelerates the degradation of m6A-modified mRNAs encoding stress-negative regulators, thereby rapidly enhancing plant salt tolerance [10]. Thus, plants have evolved a sophisticated m^6^A regulatory network that serves as a central hub for their growth, development, and adaptation to environmental stress.

Heavy metal pollution is a severe global environmental issue [11]. Cadmium (Cd) poses a significant threat to agricultural production and ecosystems due to its high toxicity and strong mobility [12]. In response to Cd stress, plants activate a series of complex molecular and physiological response mechanisms, including Cd sequestration, compartmentalization, and chelation [13]. Recent studies have begun to highlight the role of epigenetic regulation, specifically m^6^A RNA methylation, in plant responses to cadmium (Cd) stress. Evidence shows that Cd exposure induces extensive reprogramming of the m^6^A methylome in plant mRNAs. In rice, Cd stress induces cultivar-specific, large-scale rearrangements of m^6^A modifications in the root transcriptome. Differentially modified m^6^A peaks are enriched in amino acid metabolism and stress-response pathways, and correlate with altered expression of key root development genes, supporting a role for dynamic m^6^A remodeling in the epitranscriptomic regulation of Cd adaptation [14]. In barley, Cd stress induces root-wide hyper-m^6^A methylation, which positively correlates with gene expression. This reprogramming primarily targets regulatory networks rather than canonical Cd transporters (e.g., HvNramp5 and HvIRT1), revealing an indirect epitranscriptomic regulatory mode that may shape Cd tolerance through upstream signaling and transcriptional modulation [15]. In tomato roots, Cd stress globally decreases m^6^A methylation, a change negatively correlated with gene expression. Hypomethylation-associated upregulation indicates m^6^A reprogramming coordinately modulates stress pathways, including phenylpropanoid biosynthesis, glutathione metabolism, and ABC transporters. Pharmacological evidence further shows that m^6^A directly regulates Cd transporter genes *SlIRT1* and *SlNRAMP3*, confirming m^6^A-mediated post-transcriptional control as a critical mechanism in tomato Cd response [16]. These studies provide important insights into the regulatory role of m^6^A in plant cadmium (Cd) responses, indicating that the m^6^A modification network functions as a key epitranscriptomic mechanism that enables plants to adapt to Cd-contaminated environments.

Pepper (*Capsicum chinense*) is a globally important vegetable and cash crop. However, cadmium (Cd) contamination in agricultural soils seriously compromises pepper yield and quality [17]. Consequently, an in-depth understanding of the molecular mechanisms underlying pepper responses to Cd stress holds substantial theoretical value and practical relevance for developing low-Cd-accumulating or Cd-tolerant cultivars. As outlined above, m^6^A RNA modification constitutes a conserved and dynamic layer of post-transcriptional gene regulation and has been increasingly implicated in plant abiotic stress responses. However, to date, the genome-wide repertoires of m^6^A writers, erasers, and readers in pepper have not been systematically cataloged, phylogenetically classified, or functionally annotated. Comprehensive identification and annotation of m^6^A regulatory proteins represent a critical foundational step for mechanistic investigation. The aim of this study is to conduct a comprehensive analysis of m^6^A regulators (including writers, erasers, and readers) and their roles in pepper responses to Cd stress. Our work provides a foundational epitranscriptomic resource for deciphering the regulatory mechanisms underlying pepper adaptation to Cd stress.

## 2. Results

### 2.1. Genome-Wide Identification of m^6^A Regulators in Capsicum chinense (Ccm^6^As)

**Figure 1:**
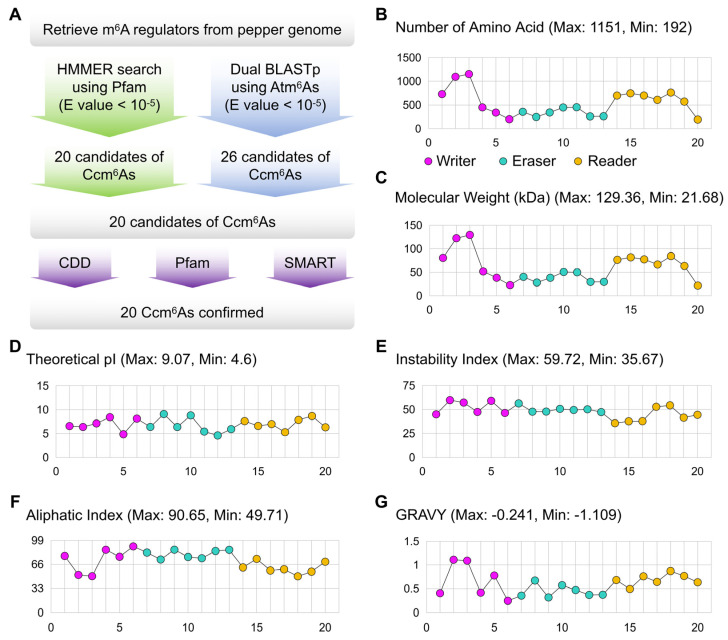
Genome-wide identification and classification of m^6^A regulatory genes in pepper. (**A**) Schematic representation of the workflow for identifying *Ccm^6^As*; (**B**–**G**) physicochemical properties of 20 *Ccm^6^As*.

To identify evolutionarily conserved Ccm^6^A regulators (including writers, erasers, and readers), a two-tiered comparative genomic approach was implemented. As shown in Figure 1A, the pepper reference genome was systematically screened using HMMER3 (v3.3.2) with curated Pfam domain models (e.g., MT-A70 for writers, AlkB for erasers, and YTH for readers). Simultaneously, reciprocal BLASTp (2.12.0+) analyses were performed using reported *Arabidopsis thaliana* m^6^A regulators (Atm^6^As) as query sequences (E-value ≤ 1 × 10^−5^). Candidate open reading frames were validated through integrated domain annotation using CDD, Pfam, and SMART databases to exclude fragmented, truncated, or non-canonical hits. This pipeline yielded a non-redundant set of 20 high-confidence *Ccm^6^A* genes: 6 writers, 7 erasers, and 7 readers. Nomenclature was standardized based on phylogenetic orthology to Atm^6^As and chromosomal/scaffold coordinates (Table S1). The deduced amino acid sequences range from 192 to 1151 residues (Figure 1B, Table S2), corresponding to predicted molecular weights of 21.68–129.36 kDa (Figure 1C, Table S2). Ccm^6^A writers exhibit marked sequence length heterogeneity, whereas Ccm^6^A readers consistently display longer polypeptides and higher molecular masses. Ccm^6^A erasers show comparatively narrow distributions in both length and molecular weight. Theoretical isoelectric points (pI) span 4.60–9.07 (Figure 1D, Table S2), with Ccm^6^A readers exhibiting higher mean pI (7.03 ± 0.01) than Ccm^6^A writers (6.91 ± 0.01) and Ccm^6^A erasers (6.64 ± 0.01). Instability Index (II) values range from 35.67 to 59.72. Notably, 17 of the 20 Ccm^6^As are predicted to be unstable (II > 40), whereas the three ECT-family readers (CcECT1, CcECT2, and CcECT5) exhibit enhanced stability (II = 35.67–37.77) (Figure 1E, Table S2). Aliphatic Index (AI) analysis reveals that Ccm^6^A erasers possess substantially higher AI values (72.59–86.04) relative to writers (49.71–90.65) and readers (49.55–73.45) (Figure 1F, Table S2), suggesting greater thermostability. All Ccm^6^A proteins display negative Grand Average of Hydropathicity (GRAVY) scores (−1.109 to −0.241), confirming their uniformly hydrophilic nature (Figure 1G, Table S2).

### 2.2. Chromosome-Scale Mapping and Collinearity Analysis of m^6^A Regulators

**Figure 2:**
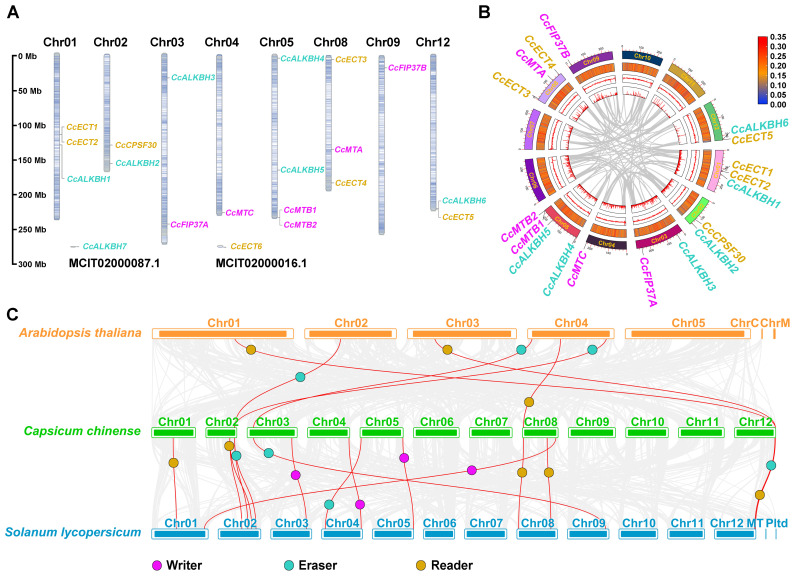
Chromosomal localization and synteny analysis of *Ccm^6^A* genes. (**A**) Chromosomal localization of the *Ccm^6^A* writer (magenta), eraser (green), and reader (yellow) genes. The scale bar on the left indicates chromosomal positions in megabases (Mb). (**B**) Intra-species collinearity analysis of the *Ccm^6^A* genes. The circular diagram, from the innermost to outermost rings, represents the N ratio, GC skew, GC content, and chromosome ideograms, respectively. Gray lines depict genome-wide colinear gene pairs excluding *Ccm^6^A* genes. (**C**) Inter-species collinearity analysis of *Ccm^6^A* genes between pepper and two reference species, tomato (*Solanum lycopersicum*) and *Arabidopsis thaliana*. Gray lines represent background colinear gene pairs across the genomes, while red lines highlight the colinear gene pairs containing *Ccm^6^A* genes.

To elucidate the genomic distribution of the 20 *Ccm^6^A* genes, chromosomal localization analysis was conducted using the high-quality reference genome of pepper. As shown in Figure 2A, a total of 18 genes were mapped to eight assembled chromosomes: Chr01 (*CcECT1*, *CcECT2*, and *CcALKBH1*), Chr02 (*CcCPSF30* and *CcALKBH2*), Chr03 (*CcALKBH3* and *CcFIP37A*), Chr04 (*CcMTC*), Chr05 (*CcALKBH4*, *CcALKBH5*, *CcMTB1*, and *CcMTB2*), Chr08 (*CcECT3*, *CcECT4*, and *CcMTA*), Chr09 (*CcFIP37B*), and Chr12 (*CcECT5* and *CcALKBH6*). The remaining two genes, *CcALKBH6* and *CcECT6*, were assigned to unanchored scaffold MTCIT02000087.1 and MTCIT02000016.1, respectively, indicating their current absence from the chromosome-level assembly. Cytogenetic mapping revealed that the majority of *Ccm^6^A* genes reside in subtelomeric or distal regions of chromosomal arms. Notably, no statistically supported gene clusters (e.g., tandem or segmental duplications) were detected, suggesting a predominantly dispersed genomic arrangement.

To investigate potential duplication events involving the *Ccm^6^A* gene family, intra-species collinearity analysis was conducted across the pepper genome. No collinear paralogous pairs were identified among the 20 *Ccm^6^A* genes, suggesting an absence of recent segmental or tandem duplication events within this gene family in pepper (Figure 2B). To assess evolutionary conservation and divergence of m^6^A regulators, we performed inter-species collinearity analyses between pepper and two reference plant species: tomato (*Solanum lycopersicum*) and *Arabidopsis thaliana*. As shown in Figure 2C, a substantial number of orthologous gene pairs were detected between pepper (13 *Ccm^6^A* genes, comprising 4 writers, 4 erasers, and 5 readers) and tomato (15 orthologs), consistent with their close phylogenetic relationship within the Solanaceae family. In contrast, only six orthologous pairs were identified between pepper (four *Ccm^6^A* genes, consisting of two erasers and two readers) and *Arabidopsis thaliana* (contributing six genes), reflecting the greater phylogenetic distance between these lineages.

### 2.3. Phylogenetic and Structural Characterization of the Ccm^6^A Writers

**Figure 3:**
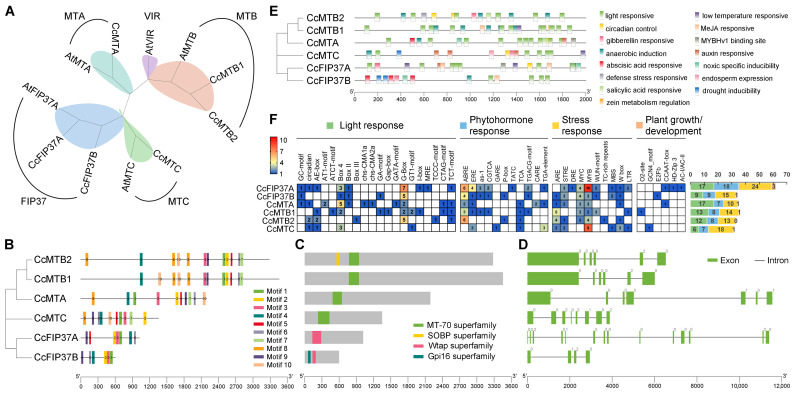
Phylogenetic relationships and domain architecture of m^6^A writer proteins. (**A**) Phylogenetic tree of the writer proteins from pepper and *Arabidopsis thaliana*; (**B**) phylogenetic tree of Ccm^6^A writer proteins with corresponding conserved motif annotation; (**C**) conserved domain architecture of Ccm^6^A writer proteins; (**D**) gene structure (exon–intron organization) of *Ccm^6^A* writer genes; (**E**) combined phylogenetic tree of Ccm^6^A writer proteins and the distribution of predicted cis-regulatory elements in their promoter regions; (**F**) quantitative distribution of cis-regulatory element categories in *Ccm^6^A* writer genes.

To resolve the evolutionary relationships within the m^6^A writer family in pepper, a neighbor-joining phylogenetic tree was reconstructed using full-length protein sequences from 11 writers, including 6 Ccm^6^A writers and 5 well-characterized m^6^A writers from *Arabidopsis thaliana*. As shown in Figure 3A, the tree resolved five well-supported clades corresponding to established functional subfamilies: MTA, MTB, MTC, FIP37, and VIR. Notably, no pepper-encoded proteins clustered within the VIR clade, suggesting either lineage-specific loss of VIR orthologs or extreme sequence divergence beyond detection thresholds in pepper. In contrast, two paralogous MTB proteins, CcMTB1 and CcMTB2, form a well-supported sister pair, indicating functional partitioning or dosage amplification.

Motif architecture analysis across the six Ccm^6^A writers (Figure 3B) revealed universal conservation of motifs 1–4, supporting deep functional conservation. CcMTB1 and CcMTB2 share identical motif composition and linear order, except for the presence of extra motif 10 in CcMTB1 and motif 8 in CcMTB2, implying post-duplication subtle divergence. CcMTA and CcMTC possess identical motif repertoires but differ in motif arrangement, implying motif shuffling as a mechanism of regulatory diversification. CcFIP37A uniquely contains motif 5, absent in CcFIP37B, potentially enabling distinct protein–protein interaction interfaces. Domain annotation (Figure 3C) confirmed that CcMTA, CcMTB, and CcMTC harbor the catalytically essential MT-A70 domain (PF05063), whereas CcFIP37A and CcFIP37B contain the WTAP homology domain (PF17098), underscoring their distinct roles as regulatory scaffolds rather than catalytic subunits in the m^6^A writer complex. Furthermore, *CcMTB2* encodes an N-terminal SOBP domain (PF15279), and CcFIP37B contains a N-terminal Gpi16 domain (PF04113), both implicated in nuclear speckle localization and assembly of RNA processing bodies. Gene structure analysis (Figure 3D) demonstrates marked architectural divergence: *CcMTA* (7 exons), *CcMTC* (8 exons), and *CcFIP37* isoforms (4–14 exons) exhibit distinct intron–exon organizations, whereas *CcMTB1* and *CcMTB2* display identical exon number and arrangement mode.

To identify candidate *cis*-regulatory elements involved in the transcriptional regulation of *Ccm^6^A* genes, we conducted in silico analysis of the 2 kb genomic sequences upstream of the transcription start site (TSS) for all 20 *Ccm^6^A* genes using the PlantCARE database. Cis-regulatory profiling classified the elements into four functional categories: light-responsive (e.g., Box 4, G-box, and TCT-motif), hormone-responsive (e.g., ABRE, ERE, and TGA-element), stress-responsive (e.g., ARE, WUN-motif, and LTR), and plant growth-/development-associated elements (e.g., GCN4_motif and HD-Zip 3) (Figure 3E). Among these, stress-responsive elements were the most significantly enriched across all writer promoters, with a mean copy number of 15.7 per promoter, followed by light-responsive (12.3) and hormone-responsive elements (9.5). Notably, elements such as ARE, MYB/MYC, ABRE, and the G-box were prominently enriched in the promoters of these genes.

### 2.4. Evolutionary and Structural Characterization of the Ccm^6^A Readers

**Figure 4:**
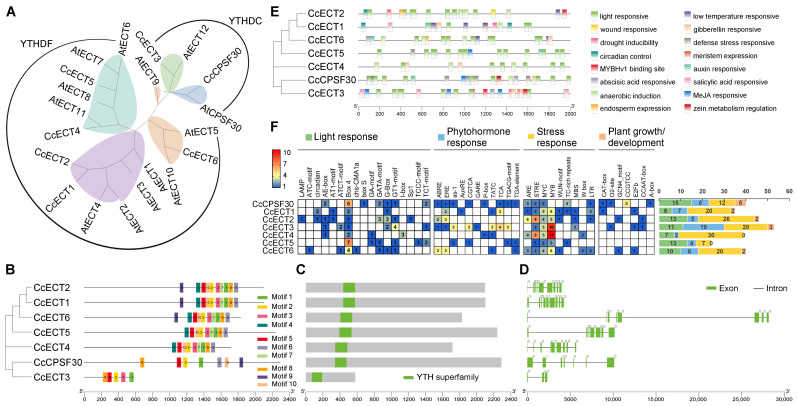
Phylogenetic relationships and domain architecture of m^6^A reader proteins. (**A**) Phylogenetic tree of the reader proteins from pepper and Arabidopsis thaliana; (**B**) phylogenetic tree of Ccm^6^A reader proteins with corresponding conserved motif annotation; (**C**) conserved domain architecture of Ccm^6^A reader proteins; (**D**) gene structure (exon–intron organization) of *Ccm^6^A* reader genes; (**E**) combined phylogenetic tree of Ccm^6^A reader proteins and the distribution of predicted cis-regulatory elements in their promoter regions; (**F**) quantitative distribution of cis-regulatory element categories in *Ccm^6^A* reader genes.

A species-comparative phylogeny of m^6^A readers was inferred from seven Cc YTH-domain proteins and 13 canonical Atm^6^A readers (Figure 4A). The tree resolved two evolutionarily conserved clades: nuclear-localized YTHDC (comprising CcECT3 and CcCPSF30) and cytoplasmic YTHDF (comprising CcECT1, CcECT2, CcECT4, CcECT5, and CcECT6). This 5:2 expansion ratio in the YTHDF clade suggests subfunctionalization or adaptive amplification of cytoplasmic mRNA fate regulation in pepper.

Motif analysis (Figure 4B) confirmed the universal presence of motifs 1, 2, 5, and 8 across all seven Ccm^6^A readers, supporting conserved m^6^A recognition capacity. Within the YTHDF clade, motif composition and linear order are fully conserved among all five members, with the sole exception of motif 9, which is present in CcECT1/2/4 but absent in CcECT5/6, potentially correlating with differential recruitment to mRNA decay versus translation activation complexes. All seven proteins encode a canonical YTH domain (PF04146) with intact aromatic cage residues essential for selective m^6^A binding (Figure 4C). Gene structure comparison (Figure 4D) reveals fundamental architectural divergence: YTHDC members (*CcECT3*, *CcCPSF30*) possess 3–7 exons, whereas YTHDF members uniformly contain 7–9 exons.

Promoter analysis (Figure 4E) revealed that stress-responsive cis-elements were the most significantly enriched class across all *Ccm^6^A* reader promoters, with a mean copy number of 19.2 per promoter, substantially exceeding those of light-responsive (10.7) and hormone-responsive (7.5) elements. Notably, elements such as ARE, STRE, and MYB/MYC were densely distributed in *Ccm^6^A* reader genes, indicating their key roles in the transcriptional and post-transcriptional coordination of stress-responsive mRNA turnover.

### 2.5. Evolutionary and Structural Characterization of Ccm^6^A Erasers

Phylogenetic reconstruction of the eraser family included 7 Ccm^6^A ALKBH proteins and 12 Atm^6^A ALKBH orthologs, revealing six well-supported subgroups (I–VI). Subgroup VI, containing two paralogous AtALKBH10 proteins (AtALKBH10A and AtALKBH10B), lacked any Cc representatives, indicating either lineage-specific gene loss or accelerated sequence divergence precluding reliable orthology assignment in pepper.

**Figure 5:**
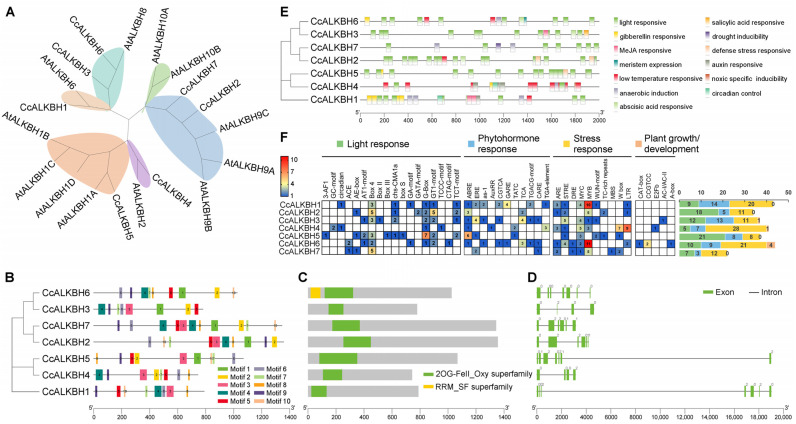
Phylogenetic relationships and domain architecture of m^6^A eraser proteins. (**A**) Phylogenetic tree of the eraser proteins from pepper and *Arabidopsis thaliana*; (**B**) phylogenetic tree of Ccm^6^A eraser proteins with corresponding conserved motif annotation; (**C**) conserved domain architecture of Ccm^6^A eraser proteins; (**D**) gene structure (exon-intron organization) of *Ccm^6^A* eraser genes; (**E**) combined phylogenetic tree of Ccm^6^A eraser proteins and the distribution of predicted cis-regulatory elements in their promoter regions; (**F**) quantitative distribution of cis-regulatory element categories in *Ccm^6^A* eraser genes.

Motif architecture across the seven Cc erasers is markedly heterogeneous (Figure 5B). All members retain the conserved motifs 5–7 and 9, but accessory motifs diverge substantially. Domain annotation (Figure 5C) confirms that all seven proteins encode catalytically competent 2OG-Fe(II) oxygenase domains (PF03171), required for Fe(II) coordination and oxidative demethylation. No premature stop codons, frameshifts, or disruptive indels were detected within these domains, supporting their functional integrity. Additionally, CcALKBH6 harbors an N-terminal RRM domain (PF0076) suggestive of RNA processing capacity. Gene structure analysis (Figure 5D) demonstrates substantial architectural diversity: *CcALKBH1* and *CcALKBH5* each contain seven exons with long introns, whereas *CcALKBH2-4* and *CcALKBH6-7* range from four to seven exons with predominantly short introns.

*Cis*-regulatory profiling (Figure 5E) revealed widespread occurrence of light-responsive, hormone-responsive, stress-responsive, and plant growth- and development-associated cis-elements across all *Ccm^6^A* eraser promoters. Notably, stress-responsive elements were the most abundant class, with a mean of 15.9 copies per promoter, followed by light-responsive (median 11.7) and hormone-responsive elements (median 8.4). Notably, MYB-/MYC-binding motifs and low-temperature-responsive (LTR) elements were significantly enriched in *Ccm^6^A* eraser promoters. In contrast, plant growth- and development-associated cis-elements were comparatively rare, exhibiting a median copy number of less than one per promoter, indicating that transcriptional regulation of these eraser genes is not predominantly governed by intrinsic developmental cues.

### 2.6. Protein–Protein Interaction (PPI) Characteristics of Ccm^6^A Regulators

To investigate the potential formation of functional complexes among pepper m^6^A regulators, we analyzed their PPI network using the STRING platform. Interaction strength is represented by a confidence score: scores < 0.4 are considered no interaction; scores between 0.4 and 0.7 indicate weak interaction; scores between 0.7 and 0.9 indicate moderate interaction; and scores ≥ 0.9 indicate strong interaction.

**Figure 6:**
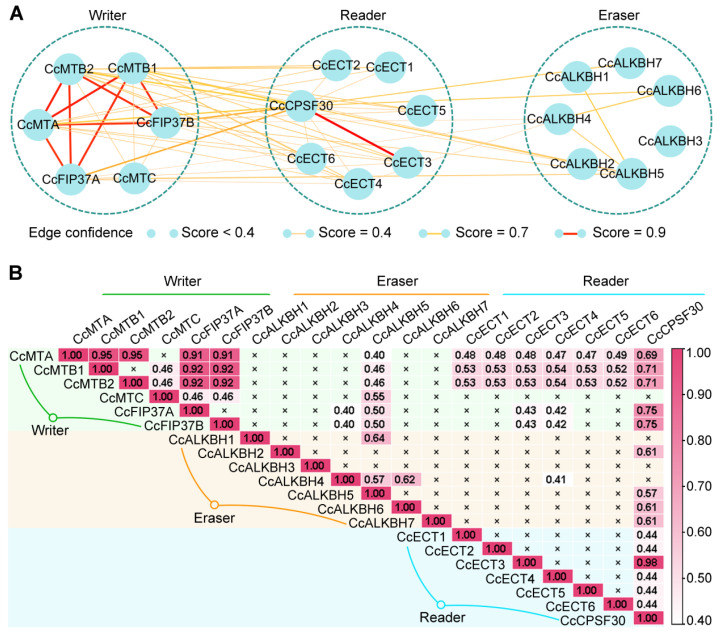
Protein–protein interaction (PPI) analysis of pepper m^6^A machinery components. (**A**) Predicted PPI network among pepper m^6^A writer, reader, and eraser proteins; (**B**) quantitative assessment of interaction confidence scores.

As shown in Figure 6A,B, protein–protein interaction (PPI) analysis was performed using the STRING platform to predict potential interactions among Ccm^6^A regulators. These interactions are computational predictions and require experimental validation. Strong predicted interaction scores (>0.9) were observed among core writer subunits (CcMTA, CcMTB1, and CcMTB2) and adaptor proteins (CcFIP37A and CcFIP37B), implying the putative formation of a conserved m^6^A methyltransferase complex, consistent with previous studies in model plants. Notably, CcMTC did not show strong predicted interactions with the above core subunits, implying that it may function independently of this core complex or participate in a distinct regulatory module.

The reader member CcCPSF30 was predicted as a critical interaction hub protein within the reader family. It not only interacts with writers (showing moderate interaction with CcMTB and CcFIP37) and erasers (showing weak interaction with CcALKBH2, 5, 6, and 7) but also serves as the key node connecting other readers (CcECT1-6). A strong interaction (score 0.98) exists between CcCPSF30 and CcECT3, suggesting they may form a stable heterodimer or functional module that cooperates in recognizing specific m^6^A modifications or recruiting downstream effectors. Apart from CcCPSF30, no direct interactions were detected among the other reader members (CcECT1-6), though they are all indirectly linked through CcCPSF30. This structure suggests that reader functions may be integrated or channeled through CcCPSF30.

Among eraser members, only sporadic and weak interactions were predicted (e.g., between CcALKBH5 and CcALKBH1/4, and between CcALKBH4 and CcALKBH6), suggesting they primarily function independently in demethylation rather than forming stable multi-subunit complexes. CcALKBH5 showed weak interactions with all six writer members (score range 0.40–0.55), implying that CcALKBH5 may have broad, dynamic functional connections with the methylation machinery, potentially participating in feedback regulation of specific m^6^A sites. CcALKBH3 exhibited no interaction with any other regulator in the network, which may indicate highly specialized functions or dependence on interaction partners not included in the current analysis.

### 2.7. Expression Patterns of Ccm^6^A Regulators Under Cadmium Stress

To elucidate the potential biological function of the m^6^A regulatory machinery in pepper under cadmium (Cd) stress, this study systematically compared phenotypic differences and the expression patterns of m^6^A regulators between a Cd-tolerant genotype (CdRes-1) and a Cd-sensitive genotype (CdSen-1). Following exposure to 4 mg/L CdCl_2_ for 5 days, pronounced phenotypic divergence was observed, particularly in root system architecture. Under control conditions, no statistically significant difference in primary root elongation was detected between CdRes-1 and CdSen-1. Upon Cd stress, root elongation in CdRes-1 was significantly reduced relative to the control; however, it remained approximately three times greater than that of CdSen-1 (*p* < 0.01). In contrast, CdSen-1 displayed near-complete inhibition of root growth (*p* < 0.001), confirming its hypersensitivity and underscoring the stronger ability of CdRes-1 to maintain root elongation under Cd stress.

**Figure 7:**
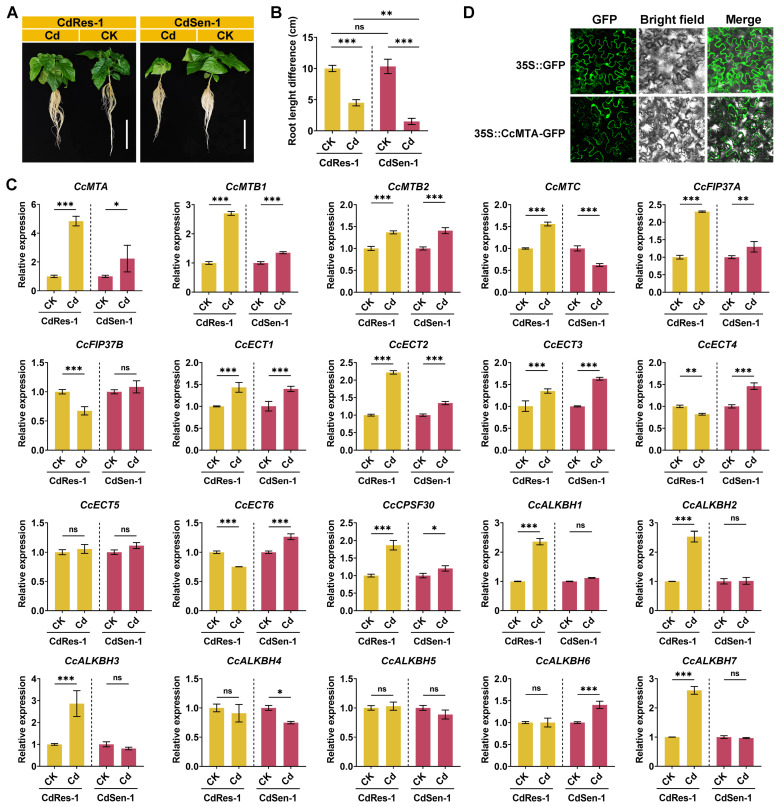
Cd-responsive expression profiles of m^6^A regulatory genes in pepper. (**A**) Phenotypic comparison of two pepper genotypes under control (CK) and cadmium (Cd) stress conditions. Scale bar = 10 cm; (**B**) root length variation in the two genotypes before and after Cd treatment; (**C**) relative transcript abundance of selected m^6^A regulatory genes quantified by qRT-PCR; (**D**) subcellular localization of the m^6^A methyltransferase CcMTA in *Nicotiana benthamiana* epidermal cells, as determined by confocal microscopy. Statistical comparisons between two experimental groups were performed using two-tailed Student’s *t*-tests. All qRT-PCR and phenotypic assays were conducted with three independent biological replicates (*n* = 3). Data are presented as mean ± standard deviation of the mean (SD); significance thresholds were set at * *p* < 0.05, ** *p* < 0.01, and *** *p* < 0.001; ns indicates no significance.

In this study, genes were considered significantly differentially expressed if they met both of the following criteria: (i) a statistically significant difference in expression (*p* < 0.05) based on qRT-PCR analysis (Figure 7C); and (ii) |log_2_(Cd/CK)| ≥ 1 in terms of qRT-PCR-measured expression levels (Table S8). Our results revealed genotype-specific transcriptional reprogramming of the m^6^A regulatory machinery (Figure 7C and Table S8). In CdRes-1, eight m^6^A regulator genes were significantly upregulated, including four erasers (*CcALKBH1*, *CcALKBH2*, *CcALKBH3*, and *CcALKBH7*), one reader (*CcECT2*), and three writers (*CcFIP37A*, *CcMTA*, and *CcMTB1*). Notably, *CcMTA* exhibited the strongest induction (log_2_FC = 2.271), suggesting a vital role in the tolerant response. In CdSen-1, only *CcMTA* was significantly induced (log_2_FC = 1.158), and its absolute expression level under Cd stress remained 2-fold lower than that in CdRes-1.

To determine the subcellular localization of CcMTA, a C-terminal CcMTA-GFP fusion construct under the control of the CaMV 35S promoter was generated and transiently expressed in *Nicotiana benthamiana* epidermal cells. Confocal microscopy revealed that the GFP signal was localized to the nucleus and cytoplasm (Figure 7D).

## 3. Discussion

N6-methyladenosine (m^6^A) is the most prevalent internal chemical modification in eukaryotic mRNA and serves as a dynamic, reversible post-transcriptional regulator of gene expression. In plants, m^6^A modulates diverse biological processes, including growth and development, signal transduction, and responses to abiotic stress, through precise control of mRNA splicing, nuclear export, stability, and translation efficiency [18,19]. This regulatory function is mediated by a conserved tripartite machinery: writer complexes (e.g., MTA/MTB/MTC core subunits and FIP37), which catalyze m^6^A deposition; eraser enzymes (primarily ALKBH family 2OG-Fe(II) oxygenases), which mediate demethylation; and reader proteins (predominantly YTH domain-containing factors), which interpret the m^6^A mark to direct downstream functional outcomes [5,20].

To date, comprehensive genome-wide inventories of m^6^A regulatory components have been established across multiple plant species: tomato (24 members) [21], soybean (42) [22], poplar (61) [23], and rice (124) [24]. Despite interspecific variation in gene family size, all identified components fall into the three canonical functional classes, including MT-A70 (writers), YTH (readers), and ALKBH (erasers), supporting the deep evolutionary conservation of the core m^6^A pathway in land plants. In this study, we identified 20 m^6^A machinery genes in the pepper (*Capsicum chinense*) genome: six writers, seven erasers, and seven readers, further corroborating the broad conservation of this regulatory system across the plant kingdom.

Phylogenetic and domain architecture analyses revealed that all pepper writer proteins contain evolutionarily conserved MT-A70 catalytic domains or WTAP-homology regulatory domains and cluster within orthologous clades (MTA, MTB, MTC, and FIP37), with strong sequence affinity to their *Arabidopsis thaliana* counterparts. Likewise, hallmark functional domains, including the 2OG-Fe(II) oxygenase domain in erasers and the YTH domain in readers, are highly conserved in the corresponding pepper proteins. These findings provide convergent evidence, at both sequence and structural levels, for the functional preservation of m^6^A catalysis and recognition mechanisms across plant lineages. Notably, the total number of m^6^A regulators in pepper (*n* = 20) closely resembles that in tomato (*n* = 24), a phylogenetically proximal Solanaceae species, but is markedly lower than in soybean (*n* = 42), poplar (*n* = 61), or rice (*n* = 124). This discrepancy is likely attributable to extensive gene loss, functional redundancy, or sub-/neofunctionalization during long-term evolution [25]. Consistent with prior reports in soybean [22], all identified pepper m^6^A regulators exhibit negative GRAVY (Grand Average of Hydropathy) values, indicative of hydrophilic, suggesting their functional engagement in dynamic, cytoplasmic or nucleoplasmic m^6^A regulatory cycles. Comparative genomic analysis further uncovered lineage-specific evolutionary events. For instance, orthologs of the *Arabidopsis* AtALKBH10A/B subfamily, known regulators of flowering time [9], are absent from both pepper and tomato genomes [21]. This implies a probable loss of these genes early in the Solanaceae lineage, potentially compensated by functional redundancy or neofunctionalization among retained erasers. Such losses may underlie species-specific adaptations, for example, divergent regulation of developmental timing or stress-responsive pathways. Conversely, pepper exhibits selective expansion within certain subfamilies. Among readers, the cytoplasm-localized YTHDF clade comprises five members (CcECT1, CcECT2, CcECT4, CcECT5, and CcECT6), whereas the nucleus-enriched YTHDC clade contains only two (CcECT3 and CcCPSF30). Similarly, the writer subfamily MTB includes two paralogs, CcMTB1 and CcMTB2. These expansions may enable enhanced regulatory granularity over cytoplasmic mRNA fate, particularly stability and translational output, potentially supporting pepper-specific physiological traits, such as fruit secondary metabolism and multifaceted environmental stress resilience [26,27].

Gene duplication remains a primary driver of functional innovation in plant gene families [28]. While *MTA* and *MTC* are typically single-copy across land plants, *MTB* has undergone amplification in several species [21,29]. Here, we report two tandemly arranged *MTB* paralogs (*CcMTB1* and *CcMTB2*) in the pepper genome, aligns with patterns of subfunctionalization or dosage-driven adaptation in epigenetics [30,31].

Previous studies have established that m^6^A writer proteins commonly assemble into a stable, multi-subunit methyltransferase complex to execute their function of site-specific m^6^A deposition on RNAs [32]. In this study, in silico PPI analysis revealed robust interactions among the pepper m^6^A writer core components (CcMTA, CcMTB1, and CcMTB2). The accessory factors CcFIP37A and CcFIP37B also displayed strong predicted interactions (score > 0.9) with these core writer subunits, indicating a potential key scaffolding role in complex assembly or localization in nuclear speckles, consistent with the functional features of their WTAP homology domain [33,34]. These findings strongly suggest the formation of a conserved, functional core methyltransferase complex capable of orchestrating either global or context-specific m^6^A reprogramming. Notably, none of these core writer components exhibited substantial predicted interaction with CcMTC, implying that CcMTC operates outside this canonical complex and likely functions within an independent, yet-to-be-characterized regulatory module. Within the m^6^A reader family, CcCPSF30 was predicted as a pivotal hub protein: it physically links the writer and eraser machineries, evidenced by reproducible weak-to-moderate interactions with CcMTB, CcFIP37, and multiple ALKBH paralogs (CcALKBH2/5/6/7). Strikingly, CcCPSF30 displayed an exceptionally high-confidence interaction with CcECT3 (STRING confidence score = 0.98), suggesting that it may serve as a central integrator for other YTH-domain-containing readers (CcECT1–6). Functional studies have established that the formation of a stable heterodimeric ECT complex enables cooperative recognition of m^6^A epitranscriptomic marks and recruits downstream effectors to modulate mRNA stability, thereby shaping the response of the transcript to environmental challenges [35]. Thus, the fact that these core components are retained and stress-responsive in pepper suggests that the m6A system is a fundamental, conserved “operating system” for post-transcriptional control. Promoter *cis*-element analysis in this study further revealed the transcriptional responsiveness of the pepper m^6^A regulatory machinery to environmental cues. Across the promoter regions of all three m^6^A regulatory components (writers, erasers, and readers), stress-, hormone-, and light-responsive elements were significantly more abundant than those associated with plant growth and development, supporting a proposed role for the m^6^A machinery as a key node in abiotic stress signal integration [21,36]. Here, we found that stress-responsive elements were the most highly enriched category in pepper m^6^A regulators. Specifically, the promoter regions of m^6^A reader genes exhibited the highest median count of such elements (19.2 per promoter), followed by eraser genes (median = 15.9) and writer genes (median = 15.7). Notably, Cd stress signals, potentially mediated by conserved MYB/MYC transcription factors binding to the enriched cis-elements in Ccm^6^A promoters, activate the writer complex (MTA-MTB-FIP37). This leads to hypermethylation of transcripts involved in metal chelation (e.g., metallothioneins) and oxidative stress defense, marking them for enhanced stability, a strategy conserved from Arabidopsis to crops like rice and tomato [14,16]. This pervasive and quantitatively robust enrichment implies that the pepper m^6^A regulatory network is transcriptionally primed for rapid, coordinated activation in response to diverse abiotic stress stimuli, thereby functioning as an intrinsic regulatory hub that integrates environmental signals with downstream gene expression reprogramming during stress adaptation.

As reported, Cd stress induces extensive transcriptome-wide alterations in m^6^A methylation patterns in plants [15]. A key physiological implication of these changes is that m^6^A serves as a dynamic, post-transcriptional regulatory layer that orchestrates the trade-off between growth suppression and stress acclimation. Specifically, the hypermethylation of genes encoding metallothioneins (MTs), glutathione S-transferases (GSTs), and peroxidases (PRXs) likely enhances mRNA stability and translation efficiency, thereby boosting the capacity for Cd chelation and ROS scavenging [37]. In tomato, m^6^A modifications on cell wall biosynthesis genes contribute to the formation of apoplastic barriers that physically restrict Cd influx into root tissues [16]. Thus, the physiological significance of m^6^A remodeling lies in its ability to coordinate the expression of growth- and defense-related transcripts, enabling plants to adapt and survive under Cd toxicity. Our study provides a tangible link by correlating the differential expression of m^6^A regulators with a distinct physiological phenotype: the maintenance of root growth under Cd stress. The tolerant genotype CdRes-1 exhibited significantly greater root elongation compared to the sensitive genotype CdSen-1 under Cd exposure. This physiological resilience was accompanied by widespread transcriptional upregulation of Ccm^6^A genes. In contrast, CdSen-1 showed severely stunted root growth and a largely muted transcriptional response of Ccm^6^A genes. This genotype-dependent divergence parallels the contrasting m^6^A modification landscapes previously reported between Cd-accumulating and Cd-tolerant rice cultivars [14].

The dynamic m^6^A landscape under Cd stress is governed by the concerted action of writer, eraser, and reader proteins, which form a sophisticated regulatory network for post-transcriptional gene regulation. The core writer complex, comprising MTA, MTB, and FIP37 in plants, is responsible for depositing m^6^A marks. Under Cd stress, the expression of writer genes (e.g., MTA and MTB) is often modulated, leading to a global or locus-specific redistribution of m^6^A peaks. For instance, in Arabidopsis, Cd-induced hypermethylation of stress-responsive transcripts is mediated by the writer machinery, marking these mRNAs for altered functional fates [38]. Of particular significance is the observation that CcMTA, the core catalytic subunit of the m^6^A writer complex, exhibited the highest magnitude of induction (log_2_FC = 2.271) specifically in the Cd-tolerant genotype CdRes-1, and its absolute expression level under Cd stress remained significantly higher than that in the Cd-sensitive genotype CdSen-1. This genotype-specific induction strongly argues against a generic stress response and instead supports a direct functional linkage between *CcMTA* expression dynamics and the Cd-tolerant phenotype. Subcellular localization analysis confirmed the enrichment of CcMTA in both the nucleus and cytoplasm, consistent with established roles of MTA orthologs in both co-transcriptional methylation within nuclear speckles and post-transcriptional mRNA modification in the cytoplasm [39,40]. Such writer induction likely targets a defined set of stress-responsive mRNAs to reinforce adaptive reprogramming, a mechanism corroborated in apple and poplar [41,42]. Moreover, site-specific methylation may modulate mRNA fate, including decay kinetics, ribosome loading efficiency, and spatial partitioning into stress granules or P-bodies, thereby enabling post-transcriptional reprogramming of cellular physiology [10,43,44].

The reversibility of m^6^A is mediated by erasers such as ALKBH9B and ALKBH10B. These enzymes fine-tune m^6^A stoichiometry on specific transcripts, thereby modulating their stability and function. In the context of Cd stress, erasers appear to selectively remove m^6^A marks from growth-promoting transcripts, potentially accelerating their decay and contributing to growth arrest [38]. Consistently, multiple m^6^A erasers (*CcALKBH1*, *CcALKBH2*, *CcALKBH3*, and *CcALKBH7*) were specifically induced in CdRes-1. The interplay between writers and erasers creates a tunable equilibrium that permits rapid transcriptional reprogramming in response to Cd.

Readers decode m^6^A marks to elicit functional outcomes, including mRNA stability, translation efficiency, and subcellular localization [2]. Under stress, readers such as ECT1 undergo liquid–liquid phase separation, sequestering stress-responsive mRNAs into stress granules or processing bodies (P-bodies) to modulate their translation [45]. This mechanism creates a reservoir of translationally silenced yet functionally primed transcripts, enabling rapid deployment upon stress relief. Notably, our study identified specific upregulation of the reader *CcECT2* in the tolerant genotype, warranting further investigation of its potential role in regulating Cd-adaptive mRNA fate decisions in pepper.

In summary, the m^6^A regulatory machinery functions as a central switchboard in plant adaptation to Cd stress. Writers mark transcripts for altered fate, erasers remove marks to reset the system, and readers execute downstream outcomes by modulating mRNA metabolism. This tripartite system enables plants to rapidly and reversibly adjust their gene expression profiles, thereby mitigating Cd toxicity.

## 4. Materials and Methods

### 4.1. Identification and Bioinformatic Characterization of the Ccm^6^A Gene Family

Coding sequences (CDS) of m^6^A regulatory genes from *Arabidopsis thaliana* were retrieved from the *Arabidopsis* Information Resource (TAIR; https://www.arabidopsis.org/) (accessed on 15 December 2025). Whole-genome protein sequences and corresponding annotation files for pepper (*Capsicum chinense*) were obtained from the NCBI Genome Database (Assembly GCA_002271895.2; https://www.ncbi.nlm.nih.gov/datasets/genome/GCA_002271895.2/) (accessed on 8 October 2025).

Gene identification was conducted using a dual-evidence strategy: (i) A local BLASTp search was performed in TBtools (v2.446), using *Arabidopsis* m^6^A regulator protein sequences as queries against the pepper proteome, yielding an initial candidate gene set; (ii) Hidden Markov Model (HMM) profiles for conserved functional domains of m^6^A regulators, specifically PF05063 (MT-A70), PF17098 (Wtap), PF15912 (VIR_N), PF04146 (YTH), and PF13532 (2OG-FeII_Oxy_2), were downloaded from InterPro (https://www.ebi.ac.uk/interpro/) (accessed on 15 December 2025). HMMER v3.3.2 was then employed to scan the pepper proteome (E-value cutoff < 1 × 10^−5^), generating an independent candidate set. Candidate genes supported by both approaches were retained for downstream analysis.

To ensure structural integrity and functional relevance, all candidate proteins were subjected to domain validation using three complementary databases (https://www.ncbi.nlm.nih.gov/cdd) (accessed on 5 January 2026): the Conserved Domain Database, Pfam, and SMART. Sequences exhibiting truncations, redundancy, or absence of canonical, full-length functional domains were excluded.

Physicochemical properties, including amino acid count (aa), molecular weight (MW), theoretical isoelectric point (pI), instability index (II), aliphatic index (AI), and Grand Average of Hydropathicity (GRAVY), were computed in batch using the ExPASy ProtParam tool (https://web.expasy.org/protparam/) (accessed on 8 January 2026).

### 4.2. Phylogenetic Analysis of the Ccm^6^A Gene Family

Multiple sequence alignment of full-length Ccm^6^A protein sequences was carried out using ClustalW (default parameters) implemented in MEGA 11. Based on this alignment, an unrooted phylogenetic tree was reconstructed using the neighbor-joining (NJ) method with 1000 bootstrap replicates; all other parameters remained at their default settings. The resulting tree was visualized, functionally annotated, and refined using the Interactive Tree of Life (iTOL) platform (https://itol.embl.de/) (accessed on 12 January 2026).

### 4.3. Chromosomal Localization and Collinearity Analysis

Chromosomal coordinates of *Ccm^6^A* genes were extracted from the pepper genome annotation and mapped onto physical chromosome positions using TBtools. A chromosomal distribution map was generated to illustrate the genomic organization of the gene family.

Guided by phylogenetic relationships (Section 4.2) and synteny-consistent chromosomal order, a systematic nomenclature was assigned to each *Ccm^6^A* gene member to reflect evolutionary orthology and positional context.

Collinearity analyses were performed using MCScanX (v1.1). Intra-genomic collinearity within the pepper genome was assessed to identify syntenic blocks. Inter-genomic collinearity was evaluated through pairwise whole-genome alignments between pepper and A. thaliana, as well as between pepper and tomato (*Solanum lycopersicum*), to detect conserved syntenic regions harboring Ccm^6^A orthologs. Alignment significance was determined using an E-value threshold of <1 × 10^−5^, and up to five top-scoring alignments per query gene were retained.

### 4.4. Comparative Analysis of Conserved Motifs, Protein Domains, and Gene Structures

Conserved protein motifs across Ccm^6^A family members were identified de novo using the MEME Suite (https://meme-suite.org/meme/) (accessed on 14 January 2026), with the following parameters: maximum number of motifs = 10; motif width range = 6–200 residues; all other settings retained as defaults.

Protein domain architecture was annotated using the NCBI Conserved Domain Database (CDD; https://www.ncbi.nlm.nih.gov/cdd) (accessed on 14 January 2026), applying an E-value cutoff of <1 × 10^−5^.

Gene structural features, including coding sequences (CDS), untranslated regions (UTRs), and exon–intron boundaries, were parsed from the pepper genome GFF3 annotation file. Integrated visualization of motif distribution, domain composition, and exon–intron architecture was generated using TBtools.

### 4.5. Cis-Regulatory Element Analysis in Promoter Regions

For each *Ccm^6^A* gene, a 2 kb genomic sequence upstream of the transcription start site (TSS) was extracted as the putative promoter region using TBtools. Cis-regulatory elements within these sequences were predicted and classified using PlantCARE (https://bioinformatics.psb.ugent.be/webtools/plantcare/html/) (accessed on 19 January 2026). Element types, frequencies, and positional distributions across the promoter cohort were summarized and graphically represented using TBtools.

### 4.6. Protein–Protein Interaction Analysis of the Pepper Ccm^6^A Gene Family

Protein–protein interaction (PPI) analysis for the Ccm^6^A family was performed using the STRING online database (https://cn.string-db.org/) (accessed on 5 February 2026) with *Capsicum chinense* set as the reference organism. Interactions with a confidence score ≥ 0.4 were retained. The resulting PPI network was visualized and further refined using Cytoscape software (v3.10.4).

### 4.7. Plant Materials and Cadmium Stress Treatment

Two contrasting pepper cultivars were employed: a cadmium-tolerant line (CdRes-1) and a cadmium-sensitive line (CdSen-1). Seeds were surface-sterilized by immersion in preheated distilled water (55 °C, 20 min), followed by thorough rinsing with sterile distilled water. Sterilized seeds were placed on double layers of moistened sterile filter paper in 10 cm Petri dishes and incubated in darkness at 28 °C for germination. Upon radicle emergence, uniformly germinated seeds were transplanted into 50-cell propagation trays filled with a sterile soilless substrate (fine sand:vermiculite:peat, 1:1:1, *v*/*v*/*v*). Seedlings were grown under controlled greenhouse conditions (16 h photoperiod, 28 °C day/25 °C night, 70 ± 5% relative humidity) until the 3–4 true-leaf stage. At this stage, morphologically uniform and vigorous seedlings were selected. Roots were gently rinsed with deionized water to remove residual substrate, and seedlings were transferred to hydroponic systems containing aerated Hoagland’s nutrient solution. Hydroponic cultivation continued under identical environmental conditions until plants reached the 8–9 true-leaf stage, at which point cadmium stress treatment was initiated.

Prior to treatment initiation, baseline phenotypic data, including digital photographs and precise measurements of primary root length, were recorded for all plants. Cadmium stress was imposed by supplementing the Hoagland’s solution with CdCl_2_ to achieve a final concentration of 4 mg L^−1^ Cd^2+^; control plants received equivalent volumes of unamended Hoagland’s solution. Treatments were maintained for five consecutive days under continuous aeration and daily solution renewal to ensure consistent Cd bioavailability and nutrient status. At harvest (day 5), primary root length was measured again. Root tissues were immediately excised, flash-frozen in liquid nitrogen, and stored at −80 °C pending downstream molecular analyses. All experiments were conducted with three independent biological replicates (*n* = 3) per cultivar–treatment combination to ensure statistical robustness and experimental reproducibility.

### 4.8. Expression Pattern Analysis of Ccm^6^A Genes

Total RNA was isolated from pepper root tissues using the RNAprep Pure Plant Kit (TIANGEN Biotech., Beijing, China), following the manufacturer’s instructions. RNA concentration and purity were quantified using a NanoDrop 2000 UV-Vis Spectrophotometer (Thermo Fisher Scientific, Waltham, MA, USA), with acceptable samples exhibiting A_260_/A_280_ ratios between 1.9 and 2.1 and A_260_/A_230_ ratios > 2.0. RNA integrity was further verified by electrophoresis on a 1% (*w*/*v*) denaturing agarose gel stained with GoldView™, confirming the presence of intact 25S and 18S ribosomal RNA bands. First-strand cDNA was synthesized from 1.5 μg of high-quality total RNA using the HiScript III All-in-One RT SuperMix for qPCR (Vazyme Biotech., Nanjing, China), including gDNA wiper mix to eliminate genomic DNA contamination.

Gene-specific primers for all Ccm^6^A family members and the reference gene were designed using Primer 6.00 software to ensure specificity, amplicon length (150–300 bp), and absence of secondary structures or primer-dimer formation (see primer sequences in Table S6, and see melting curve in Figure S1). Quantitative real-time PCR (qRT-PCR) was performed in triplicate biological replicates (*n* = 3) using the ChamQ Universal SYBR qPCR Master Mix (Vazyme Biotech., China) on a Bio-Rad CFX96 Real-Time PCR Detection System (Bio-Rad Laboratories, Singapore). The thermal cycling profile comprised an initial denaturation at 95 °C for 30 s, followed by 40 cycles of 95 °C for 10 s and 60 °C for 30 s, with fluorescence acquisition at the end of each extension step. Melting curve analysis (65–95 °C, increment 0.5 °C/5 s) confirmed amplification specificity. The pepper ACTIN gene (GenBank accession: GQ339766.1) was selected as the internal reference. Relative transcript abundance was calculated using the 2^−ΔΔCt^ method. Graphical visualizations were conducted using GraphPad Prism (v8.0.2).

### 4.9. Subcellular Localization Analysis

The full-length coding sequence (CDS) of *CcMTA* was amplified from pepper cDNA using high-fidelity 2 × Phanta Max Master Mix (Dye Plus) (Vazyme Biotech.), with gene-specific primers incorporating appropriate restriction sites (see primer at Table S7). The purified PCR product was cloned into the binary vector pCambia1300 downstream of the CaMV 35S promoter and in-frame with the N-terminal GFP tag, yielding the transient expression construct pCambia1300-35S::CcMTA-GFP. Correct insertion and reading frame were confirmed by Sanger sequencing. The recombinant plasmid was transformed into *Agrobacterium tumefaciens* strain GV3101. Single colonies were cultured overnight in LB medium supplemented with rifampicin (50 μg mL^−1^) and gentamicin (10 μg mL^−1^), and the bacterial suspension was prepared in infiltration buffer (10 mM MgCl_2_, 10 mM MES, pH 5.6, and 150 μM acetosyringone), adjusted to an OD_600_ of 1.0. Five-week-old *Nicotiana benthamiana* plants were infiltrated on the abaxial leaf surface using a 1 mL needleless syringe. Infiltrated plants were incubated in darkness for 24 h to suppress defense responses, then transferred to standard growth conditions (16 h photoperiod, 25 °C day/22 °C night) for 48 h to allow for optimal fusion protein expression.

Leaf disks (10 mm diameter) were excised from infiltrated zones, mounted in water on glass slides, and immediately imaged using a confocal laser scanning microscope (Leica SP8, Wetzlar, Germany) equipped with a 488 nm argon laser for GFP excitation and a 500–550 nm bandpass emission filter.

## 5. Conclusions

In the present study, we conducted a genome-wide identification and systematic bioinformatic characterization of the regulatory machinery in pepper, revealing its composition, genomic distribution features, synteny conservation patterns, and evolutionary relationships across diverse plant lineages. Furthermore, in silico PPI analysis supports the formation of a functional m^6^A methyltransferase complex containing CcMTA, CcMTB1, and CcFIP37A. Critically, comparative analysis between the Cd-tolerant genotype CdRes-1 and the Cd-sensitive genotype CdSen-1 uncovered genotype-dependent expression patterns of the m^6^A regulators in pepper in response to Cd stress. Our findings provide a foundational epitranscriptomic resource for an in-depth understanding of the regulation of heavy-metal responses in pepper.

## Data Availability

The datasets presented in this study can be found in online repositories. The names of the repository/repositories and accession number(s) can be found in the article/Supplementary Materials.

## Supplementary Materials

The following supporting information can be downloaded at: https://www.mdpi.com/article/10.3390/ijms27094110/s1.
