# Supplementary material for: Comprehensive Genomic Characterization of m6A Methylation Machinery and Its Cadmium-Responsive Expression Profiles in Pepper (Capsicum chinense)
Source: Int J Mol Sci. 2026 May 4;27(9):4110. doi: 10.3390/ijms27094110 (PMC13163424; doi:10.3390/ijms27094110)
Supplement: Supplementary file 1 [file ijms-27-04110-s001.zip › Supplementary figure_Proof.pdf]

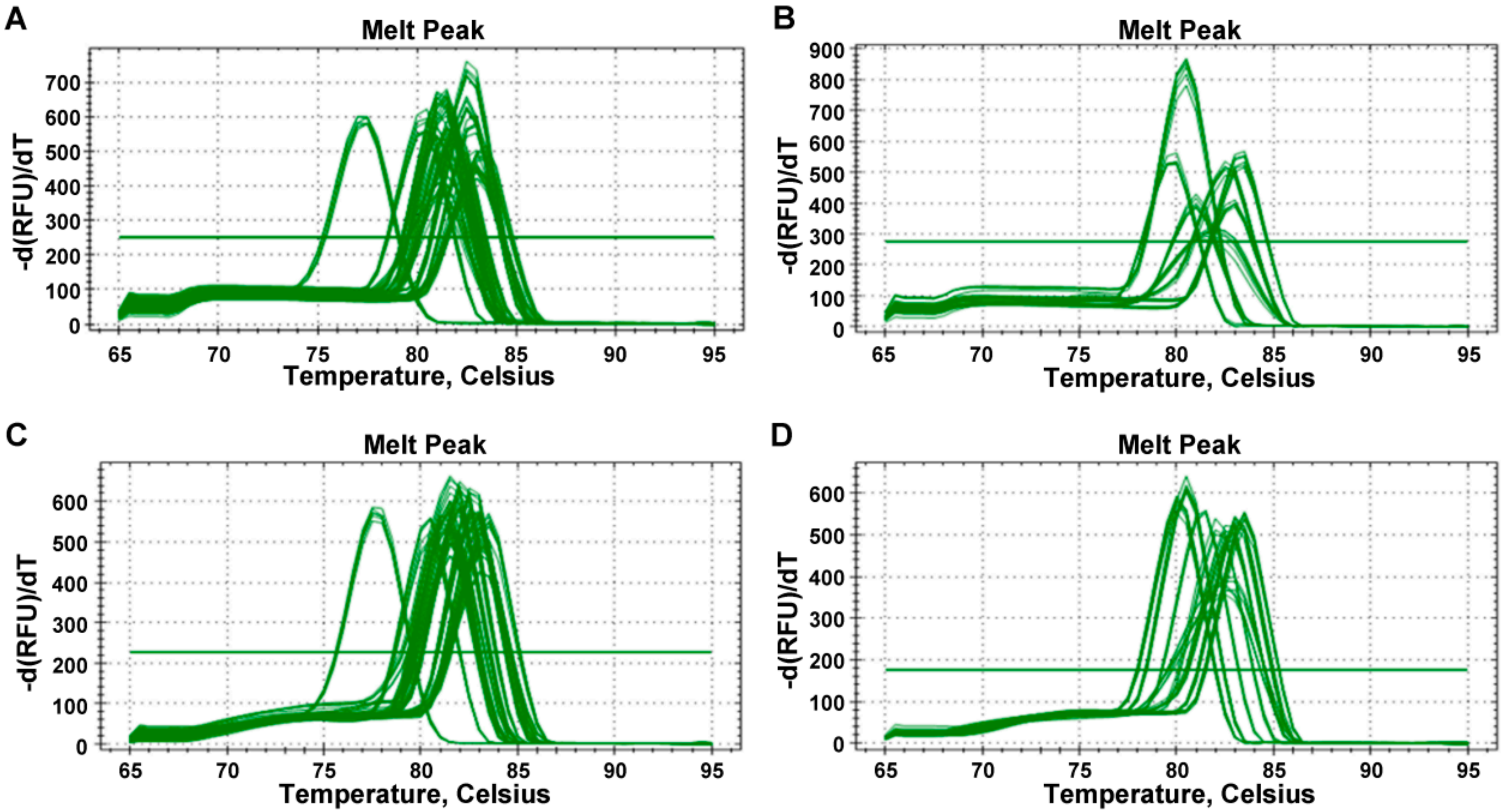

Figure S1. Melting curve confirming single-peak amplification and high primer specificity. (A-B) Melting curve of target genes in CdSen-1; (C-D) melting curve of target genes in CdRes-1.
